# Emerging and Endemic Infections in Wildlife: Epidemiology, Ecology and Management in a Changing World

**DOI:** 10.3390/pathogens13060513

**Published:** 2024-06-18

**Authors:** Andrew W. Byrne, Eric R. Morgan

**Affiliations:** 1 One-Health Scientific Support Unit, Department of Agriculture, Food and the Marine, Agriculture House, D02 WK12 Dublin, Ireland; 2 School of Biological Sciences, Queen’s University Belfast, Belfast BT9 5DL, UK; eric.morgan@qub.ac.uk

## 1. Introduction

The importance of gaining a greater understanding of the infectious diseases of wild animal populations and the impact of emerging and re-emerging pathogens has never been more sharply in focus than in the current post-COVID-19 world. The zoonotic origin of the pandemic [1], its links with damaging human impacts on nature [2] and increasing public interests in these links [3] provide both an urgent need and a timely opportunity to engage more deeply in the topic of this Special Issue. Pathogens of wildlife populations that spill over to humans (zoonoses) and domesticated animals account for a significant proportion of the most pressing emerging, reemerging and endemic diseases impacting human and veterinary health globally [4]. Although perhaps obvious that new diseases in humans are most likely to come from animals, especially wildlife, the risk of emergence is higher where the reporting effort is low [5], while effective diagnosis is key to discovering spillover events. Apart from the appearance of new diseases from wildlife, the presence and spillback of antimicrobial and antiparasitic resistance in wildlife populations are an additional source of concern. Reliance on antimicrobials in human medicine and food production has led to the widespread dissemination of resistant genes into the environment [6], and the place of wild animals in the fate of this ‘resistome’, including the potential to combine resistant genes into emerging pathogens, is highly uncertain [7].

It is essential that we place greater emphasis on identifying pathogens within wildlife populations, engaging in active and passive surveillance, gaining a greater understanding of the ecology and epidemiology of the disease and developing early detection and warning systems and appropriate control approaches from local to international scales. Such monitoring and control efforts will benefit from being integrated into a broader One Health strategy, where the health of the wildlife populations themselves, as well as the health of the broader ecosystem, is considered. This Special Issue assembles contributions relating to the identification, monitoring, ecology and control of emerging and endemic wildlife diseases. The range of pathogens is broad, spanning bacterial (*Mycobacterium tuberculosis* complex, *Coxiella* sp., tularemia), viral (herpes, rabies, rabbit hemorrhagic disease) and parasitological (*Echinococcus* and other helminths, toxoplasmosis) pathogens of wildlife hosts. These pathogens carry known risks to veterinary and human population health and circulate in wildlife populations from the Arctic to the tropics. All are subject to global change, including climatic, environmental and societal factors, and the papers in this Special Issue specifically consider how these factors influence the epidemiology of such infections and challenges around their detection and control.

## 2. An Overview of the Published Articles

Brett Gardner and colleagues (contribution 1) considered the role of gulls in the transmission of the emerging bacterial pathogen *Coxiella burnetti* in Australian fur seals. High bacterial loads were found in birds feeding on the placentas of seals but not in birds on a different island without seals, indicating the potential for gulls to act as mechanical vectors of infection and dissemination to neighboring islands. This study also highlights the gap between finding a pathogen in a wild animal species and understanding its role in maintenance and spread, which typically requires significant ongoing research effort.

The article by Buhler et al. (contribution 2) continued this theme, examining the roles of climate and rodent populations in the dynamics of tularemia, another bacterial zoonosis. In the Arctic, rates of exposure in foxes were related to the abundance of lemmings and voles, which experience strong interannual population cycles, and were also influenced by climatic variables. Climate warming is especially extreme in the Arctic with changes to snow cover, precipitation, bird migration and biting insect populations. This paper showed the complexity of interacting factors that could be implicated in disease emergence with environmental change. Consequently, it can be difficult to know how best to represent and track changes in disease risk over time, and foxes could prove a useful sentinel given their position as a predator and scavenger of both rodents and birds and the role of fox hunting as a source of animals for testing.

The theme of wildlife sentinels of infection continued in Martini et al. (contribution 3), this time for the zoonotic tapeworm *Echinococcus multilocularis* in Europe. Despite being an invasive species on the continent, the musk rat shows high infection prevalence and intensity and can be an effective sentinel for infection in foxes and for zoonotic risk to humans. The role of this species in disease dynamics is less clear, however, since it is localized to riparian environments and makes up a much smaller proportion of fox diets than smaller rodents such as voles. This demonstrates the general point that wildlife species with the highest prevalence of infection are not necessarily those most implicated in pathogen transmission.

Accurate detection of infection in wild animals is a significant challenge whether for monitoring or research and this problem was addressed by Clarke et al. (contribution 4). African buffaloes are important maintenance hosts of bovine tuberculosis (bTB), with consequences for wildlife populations and spillover to domestic ruminant livestock. While testing buffalo before translocating them reduces this risk, false positive tests lead to unnecessary culling, and the paper seeks to avoid this scenario by optimizing the use of existing tests to improve the specificity. Serial testing using the interferon-gamma (IFN-γ) release assay (IGRA) and the IFN-γ-inducible protein 10 release assay (IPRA) achieved very high specificity and avoided false positives in animals from historically bTB-free herds.

The quest to refine the diagnosis of infections in wild animals was continued by Tsai et al. (contribution 5), who reported a novel gamma herpes virus strain in badgers in England. Despite typically low genetic variation in herpes viruses within host species, this novel variant has emerged to become common in badgers and shows higher virulence, as well as increasing bacterial coinfections. The processes underlying the emergence of the increased virulence of viruses in wildlife remain highly debated, and their relevance to One Health is obvious, further highlighting the importance of continuing to monitor viral populations in wild animals and to be alert to changing genotypes.

In contribution 6, Gumbo et al. returned to the theme of the accurate detection of *Mycobacterium bovis* infection, this time in lions. Although maintained in buffalo, the infection can spill over to lions through predation and then be transmitted within prides, with negative consequences for health and fitness. The authors adapted commercial bTB tests to blood samples from lions, with promising results, which will help to detect and manage the disease. More generally, this shows the value of adapting commercially available tests designed for domestic animals to wild species, with appropriately rigorous evaluation, to support the study and monitoring of pathogen dynamics.

Detection of viral strains and the epidemiology of pathogen maintenance in wild animal communities were brought together in contribution 7. Garcés-Ayala and colleagues described cases of rabies virus infection in cougars in Mexico, which were confined to northern states in which canine rabies is no longer recorded. They consider that rabies is maintained in those areas by skunks; however, cougars could act as vectors, as they are at the top of the food chain and potential bridge hosts into domestic animals and humans through attacks.

The final primary research paper in this Special Issue, contribution 8 by Didowska et al., addressed the issue of the accurate diagnosis of tuberculosis in wild species. Infection in European bison was diagnosed using an interferon release assay, with better results than using the intradermal tuberculin test and strong agreement with the necropsy findings. A test with high specificity as well as high sensitivity is especially important in this species, which is near threatened with small local population sizes, such that the unnecessary culling of uninfected individuals must be strenuously avoided.

Further contributions surveyed existing knowledge on some key and emerging topics. In contribution 9, Byrne et al. provided an update on rabbit hemorrhagic virus 2 in wild hares in Ireland, a disease of concern from a conservation perspective whose epidemiology is only beginning to be understood. Contribution 10 by Brown and Morgan analyzed the existing information on gut helminth diversity in deer in Europe, noting the importance of contact with livestock pasture and the lack of evidence for the maintenance of cattle and sheep parasites in deer populations without such contact. Bokaba et al. closed out the Special Issue with contribution 11, collating information on *Toxoplasma gondii* in wild animals in Africa, including in wild felids such as lions, and calling for a more advanced understanding of its epidemiology in African ecosystems.

## 3. Conclusions

This compilation of articles demonstrates—through detailed case studies—three main points. Firstly, the dynamics of infectious diseases in wild animal populations are complex, frequently involving transmission in multi-host communities and subject to external forcing from multiple sources, especially environmental change. Secondly, the accurate detection of pathogens in wild animals can be difficult but is crucial for proper monitoring and management and can be greatly assisted by the careful adaptation of tests developed for use in domestic animals. Finally, diseases in wild animal populations rarely stand still, and ongoing active monitoring is needed to keep track of their epidemiology and distribution geographically and among hosts and evolutionary changes, including shifts in virulence and potentially host range.

The drivers of disease emergence in wildlife and the spillover to domestic animals and humans have received increasing attention over the past few decades; however, efforts to embed this understanding into a One Health perspective and holistic policy and management responses have had patchy success. The slow pace of change towards these objectives, and the potential consequences for averting or, at least, anticipating spillover of diseases of high concern, have been emphasized, presciently [8]. A major challenge arises from the ability of system level changes to perturb disease dynamics and both generate and conceal shifts in their epidemiology and in the perceived effects of management [9]. This highlights the need for concerted and coordinated efforts to collate new and existing data streams, coupled with the application of novel analytical approaches [10,11]. Interactions between contrasting influences on disease are likely to be common, hard to measure, and harder still to predict under future conditions in a rapidly changing world [12,13]. The robust science, contextual thinking, and community building exemplified in this Special Issue gives hope that these challenges are being addressed with vigor and innovation.
